# Propensity score analysis of triglyceride-glucose index in newly diagnosed patients with essential hypertension as a predictor of microalbuminuria

**DOI:** 10.3389/fendo.2026.1737230

**Published:** 2026-03-09

**Authors:** Nuoni Wang, Shihao Liu, Wei Wang, Yicheng Zou, Liangqing Ge, Sulan Huang

**Affiliations:** 1 Department of Cardiac Electrophysiology, Changde Hospital, Xiangya School of Medicine, Central South University (The First People’s Hospital of Changde City), Changde, China; 2 Department of Science and Education, Changde Hospital, Xiangya School of Medicine, Central South University (The First People’s Hospital of Changde City), Changde, China; 3 Department of Cardiology, Changde Hospital, Xiangya School of Medicine, Central South University(The First People’s Hospital of Changde City), Changde, China

**Keywords:** essential hypertension, insulin resistance, microalbuminuria, propensity score analysis, the triglyceride-glucose index

## Abstract

**Background:**

The Triglyceride-Glucose (TyG) index has emerged as a potential predictor for microalbuminuria (MAU) in patients with essential hypertension. This study aims to assess the TyG index as a predictor of MAU in newly diagnosed hypertensive patients, using propensity score matching (PSM) to control for confounding factors.

**Methods:**

A cohort of 2,052 newly diagnosed hypertensive patients from Changde Hospital, China (January 2020 to December 2024), was analyzed. The TyG index cutoff value was determined by receiver operating characteristic (ROC) analysis, with a value of 9.125. PSM was employed to balance baseline differences between low and high TyG index groups, and logistic regression models were used to analyze the association between TyG index and MAU. Subgroup analyses and sensitivity analyses were conducted to evaluate the robustness of the findings.

**Results:**

In the final cohort, 2,052 patients were divided into two groups based on the optimal TyG index cutoff value of 9.125. After propensity score matching (PSM), the high TyG index group (≥9.125) exhibited significantly higher rates of MAU compared to the low TyG index group (<9.125). In the adjusted models, the odds ratio (OR) for MAU in the high TyG index group was 2.37 (95% CI 1.73–3.26). The analysis revealed a non-linear, L-shaped association between TyG index and MAU, with a marked increase in the prevalence of MAU in the high TyG group. Sensitivity analyses, including inverse probability treatment weighting (IPTW), reinforced these findings, with the high TyG index group consistently showing a higher risk of MAU across both original and matched cohorts.

**Conclusions:**

The TyG index is a simple and accessible biomarker for predicting MAU in newly diagnosed hypertensive patients, providing valuable insight for early detection of kidney damage in this population.

## 1 Introduction

Microalbuminuria (MAU) is an early marker of kidney damage and a strong predictor of cardiovascular events and chronic kidney disease progression in hypertension (1, 2). Early identification of MAU at the time of hypertension diagnosis enables timely initiation of renin–angiotensin–aldosterone system (RAAS) blockade, metabolic optimization, and lifestyle interventions, which together can attenuate renal decline and reduce cardiovascular risk (1–3).

Insulin resistance (IR) contributes to hypertension-related microvascular injury through endothelial dysfunction, oxidative stress, and podocyte damage (4–6). The triglyceride-glucose (TyG) index, calculated from fasting triglyceride and glucose levels, has emerged as a reliable and easily accessible surrogate marker of IR, demonstrating a strong correlation with gold-standard hyperinsulinemic-euglycemic clamp measurements (5–7). Growing evidence supports its utility in predicting a range of cardiometabolic disorders, including arterial stiffness, incident hypertension, chronic kidney disease, and cardiovascular mortality, even among non-diabetic individuals (8–11).

Early detection of MAU in patients with essential hypertension is critical for timely intervention to mitigate renal and cardiovascular risks. However, the association between insulin resistance, as estimated by the TyG index, and MAU in the newly diagnosed, treatment-naïve hypertensive population remains inadequately characterized (12, 13). Previous studies often included patients with established diabetes, prior antihypertensive exposure, or advanced cardiometabolic comorbidities, which may confound the true relationship between the TyG index and early renal injury (14). Investigating this association specifically at the point of diagnosis is essential, as it eliminates the confounding effects of antihypertensive medications and allows for early risk stratification before significant renal decline occurs (2, 15, 16).

Clarifying whether the TyG index-MAU association is linear or exhibits a threshold—and establishing an actionable cutoff—could inform targeted MAU screening and follow-up strategies at the first hypertension diagnosis. Building on this rationale, we investigated the association between the TyG index and MAU in a contemporary cohort of newly diagnosed, treatment−untreated essential hypertension patients, excluding diabetes and established chronic kidney disease (CKD). Given the retrospective cohort design of this study, inherent baseline differences between patients with low and high TyG index levels were expected and observed (e.g., in age, body mass index (BMI), blood pressure, and lipid profiles). To mitigate confounding by these non-randomly distributed covariates and approximate a randomized comparison, we employed propensity score matching (PSM) (17). This method balances measured confounders between exposure groups, thereby strengthening causal inference regarding the association between the TyG index and MAU in the absence of prospective randomization. The use of PSM, along with sensitivity analyses using inverse probability of treatment weighting (IPTW), aligns with contemporary methodological standards for observational studies aiming to reduce selection bias and enhance the validity of estimated effects.

## 2 Subjects and methods

### 2.1 Study population

This study included newly diagnosed patients with essential hypertension who were enrolled at the Cardiovascular Department of Changde Hospital, China, between January 2020 and December 2024. An ongoing nonselective collection of data on hypertension patients over the age of 18 years was conducted at Changde Hospital, with identifying information about patients removed. Data were collected using semistructured questionnaires. The training was provided to the data collectors on the study objectives, procedures, confidentiality, respondents’ rights, and informed consent. The data collection process was supervised intensively. Written informed consent was obtained from patients agreeing to participate in the study. The institutional ethics committee clearance was obtained. The data were excluded if more than 10% of the data were missing. Missing data for records with 10% of missing variables were handled using multiple imputations. With multiple imputation, missing observations can be replaced by plausible values drawn from the posterior predictive distribution based on the observations (18).

**Figure 1:**
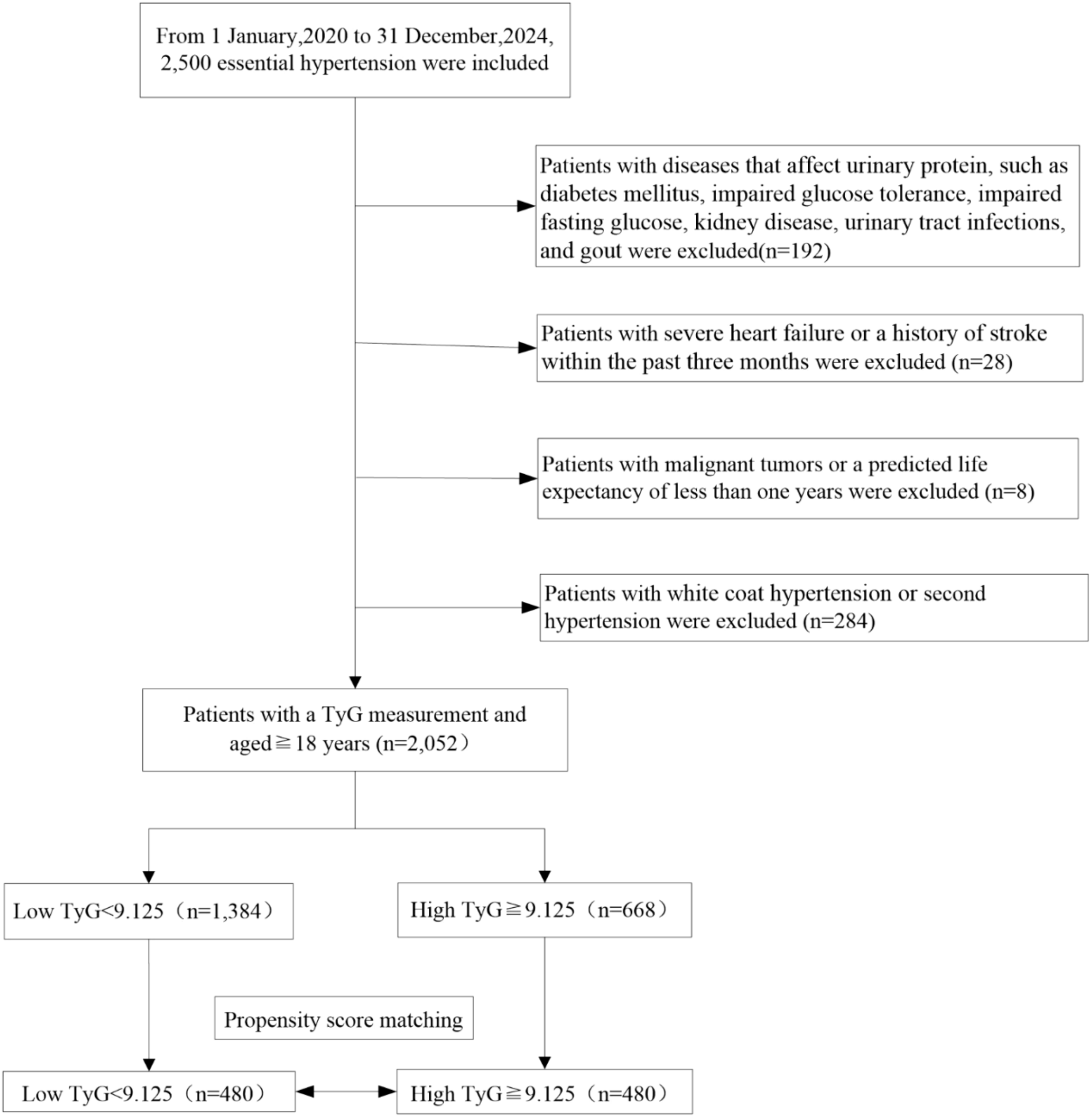
Flowchart of the inclusion and exclusion process.

Inclusion criteria:1) Patients with a clear diagnosis of hypertension, who, before treatment, have a mean blood pressure measured on three non-consecutive days, with systolic blood pressure ≥140 mmHg and/or diastolic blood pressure ≥90 mmHg. 2) Aged 18 years or older. 3) All patients must have never used antihypertensive medication before inclusion. Exclusion criteria: 1) Diseases that affect urinary protein, such as diabetes mellitus, impaired glucose tolerance, impaired fasting glucose, kidney disease, urinary tract infections, and gout. 2) White coat hypertension or secondary hypertension. 3) Severe heart failure or a history of stroke within the past three months. 4) Malignant tumors or a predicted life expectancy of less than one year. Then, 2052 participants were selected as the final analysis sample (**Figure 1**).

### 2.2 Data collection

All measurements were completed within 48 hours of admission under standardized conditions. Baseline anthropometrics (age, gender, height, weight, and smoking) were extracted from medical records. BMI was calculated as weight(kg)/height(m)^2^. Laboratory analysis required 12h of overnight fasting before venous blood collection for tests including red blood cell count (RBC), white blood cell count (WBC), hemoglobin, low-density lipoprotein cholesterol (LDL-C), high-density lipoprotein cholesterol (HDL-C), total cholesterol (TC), triglycerides (TG), and fasting blood glucose (FBG). The 2-hour postprandial blood glucose (2h-PG) levels were measured following a 75g oral glucose tolerance test. Shenzhen New Industries Biomedical Engineering Co., Ltd., Shenzhen, China) was used to determine the plasma aldosterone concentration and plasma renin activity (PRA). Participants abstained from caffeine, alcohol, smoking, and vigorous exercise for 12 hours before phlebotomy.

The calculation of TyG index=Ln [fasting triglyceride(mg/dL) ×fasting glucose (mg/dL)/2] (11, 19).

### 2.3 Blood pressure measurement

BP measurements for each patient were obtained two or more times on separate days after at least 10 minutes of rest at the cardiology outpatient clinic or ward. Patients with high clinical BP (mean SBP ≥140 mmHg and/or mean DBP ≥90 mmHg) underwent 24-hour ABPM (20). Patients aged>18 years who had been examined in detail upon admission to our clinic and followed with the diagnosis of essential hypertension were evaluated for this study (14, 20). A 24-hour ABPM (Sun Tech Medical Inc., Morrisville, NC, USA), which recorded BP and pulse rate in the non-dominant arm at 30-minute intervals in the daytime and at 60-minute intervals at nighttime, was performed in patients with high BP. The daytime was referred to the time interval between 06:00 A.M. and 10:00 P.M., and the nighttime was referred to the time interval between 10:00 P.M. and 06:00 A.M. In patients whose acceptable measurements in daytime and nighttime were below 80%, a second 24-hour ABPM was conducted (14, 20).

### 2.4 Definition of newly diagnosed patients with essential hypertension

Newly diagnosed patients with essential hypertension refer to individuals who have recently received a confirmed diagnosis of essential hypertension, characterized by persistently elevated blood pressure (≥140 mmHg systolic and/or ≥90 mmHg) measured on at least two separate occasions, and have not yet initiated any long-term antihypertensive pharmacotherapy or structured lifestyle intervention for blood pressure control. This definition typically excludes individuals with secondary hypertension, prior use of antihypertensive medications, or established cardiovascular or renal complications directly attributable to hypertension (14, 20).

### 2.5 Propensity score matching

PSM was employed because it was difficult to achieve complete stochasticity during patient screening. PSM balances the selection bias and underlying confounding factors. To control for confounding factors like age, gender, and obesity, the following approach was used: Matching variables: BMI, age, gender, smoking, estimated glomerular filtration rate(eGFR), uric acid (UA), TC, LDL-C, office systolic blood pressure (OSBP), office diastolic blood pressure (ODBP), and, heart rate (HR). Methodology: 1) Propensity scores were generated using logistic regression (high TyG index vs low TyG index as the outcome). 2) 1:1 nearest-neighbor matching was performed with a caliper width of 0.05 standard deviations (SD) of the logit propensity score. 3) The R package MatchIt (v4.2.1) was used for implementation. 4) Covariate balance was confirmed using standardized mean differences (<0.20).

### 2.6 MAU collection and definition

A spot urinary sample was taken for urine routine, microscopy examination, and estimation of urinary albumin creatinine excretion. The urine albumin reagent was used for the quantification of spot urinary albumin by the turbidimetric method on the Beckman Coulter clinical chemistry Auto Analyzer. Urinary creatinine was measured by the Jaffe kinase method. The calculated ratio between urinary albumin and creatinine was taken for urine albumin creatinine ratio (UACR) determination (21, 22). Patients with a significant rise in the excretion of UACR within the particular range of 30–299 mg of albumin per g of creatinine or a urinary albumin excretion rate (UAE) of between 30–299 mg per 24 hours were regarded as having MAU, in accordance with Kidney Disease: Improving Global Outcomes (KDIGO) guidelines (21, 22). This study excluded patients with clinical albuminuria (UACR ≥300 mg/g).

### 2.7 Statistical analyses

Variables with continuous values were reported as averages ± SD or medians (interquartile range). Based on the normality of the data distribution, Student’s t- or Mann-Whitney U-tests were used for comparisons. Categorical variables were analyzed using the chi-square test and reported as case numbers (%).

By calculating the maximum Youden index for MAU, the optimal cutoff value of the TyG index was determined using ROC analysis, in which the Youden index was equal to the sum of sensitivity and specificity minus 1. The best cutoff value of the TyG index was 9.125, with 39.9% sensitivity, 72.9% specificity, 52.1% positive predictive value, 62.1% negative predictive value, and 0.182 Youden index. The patients were divided into two groups according to the TyG index cutoff value.

Restricted cubic spline (RCS) analysis was employed to explore the potential non-linear relationship between TyG-index and MAU. Restricted cubic spline (RCS) functions with four knots placed at the 5th, 35th, 65th, and 95th percentiles of the TyG index were incorporated into multivariable logistic regression models to flexibly characterize the dose–response relationship between the TyG index and MAU; nonlinearity was evaluated using a likelihood ratio test comparing the model including the spline terms with the corresponding linear-only model (*P* for nonlinearity). Univariate and multivariate logistic regression analyses were performed to evaluate the prognostic relevance of the TyG index. After considering clinical significance, the final adjusted model retained the covariates that caused significant changes in the effect estimate of >10% (*P* < 0.05) (23). The propensity score (PS) was estimated to perform sensitivity analyses to calculate IPTW. In this example, 1/PS is the weight of high TyG index, whereas 1/(1–PS) corresponds to the weight of low TyG index. The IPTW model was then used to create a weighted cohort. Sensitivity analysis was conducted using two relationship inference models in the original and weighted cohorts, respectively (24, 25). Next, we analyzed the modifications and interactions of the subgroups based on the likelihood ratio test.

The statistical analyses were performed using IBM SPSS Statistics for Windows, version 26.0 (IBM Corp., Armonk, NY, USA), R (http://www.R-project.org, The R Foundation), and Empower Stats software (www.empowerstats.com, X&Y Solutions, Inc., Boston, MA, USA).

## 3 Results

### 3.1 Baseline characteristics

**Table 1** shows the baseline characteristics of the 2,052 study individuals according to the TyG index groups. The average age was 46.8 ± 7.8 years, and the proportion of males was 62.2%. Patients in the higher TyG index group were younger and tended to be male, and had a higher BMI level. They had higher levels of eGFR, UACR, UA, TC, LDL-c, 2hPG, homocysteine (Hcy), platelets, and PRA but lower levels of creatinine and HDL-C than those with a low TyG index. In addition, they also had higher levels of OSBP, ODBP, HR, 24-hour systolic blood pressure(24hSBP), 24-hour diastolic blood pressure(24hDBP), daytime systolic blood pressure (DSBP), daytime diastolic blood pressure (DDBP), night-time systolic blood pressure (NSBP), and night-time diastolic blood pressure (NDBP) than those in the low TyG index group.

**Table 1:** Baseline characteristics of participants according to TyG index group.

TyG index group	Total	Low TyG index	High TyG index	SMD	*P*-value
N	2052	1384	668		
BMI (kg/m2)	25.88 ± 3.41	25.33 ± 3.35	27.04 ± 3.25	0.52	<0.001
Age (years)	46.83 ± 7.78	47.43 ± 7.74	45.60 ± 7.73	0.24	<0.001
Gender				0.15	0.002
female	776 (37.82%)	556 (40.17%)	220 (32.93%)		
male	1276 (62.18%)	828 (59.83%)	448 (67.07%)		
Smoking				0.04	0.356
no	1748 (85.19%)	1172 (84.68%)	576 (86.23%)		
yes	304 (14.81%)	212 (15.32%)	92 (13.77%)		
eGFR (ml/min/1.73m2)	102.06 (88.72-116.28)	97.29 (86.33-109.77)	112.32 (97.16-125.15)	0.58	<0.001
Creatinine (umol/L)	71.00 (59.00-80.00)	71.50 (59.10-81.00)	69.00 (59.00-78.00)	0.17	<0.001
UACR (mg/g)	25.80 (11.70-43.10)	23.80 (10.70-38.50)	32.60 (13.95-53.60)	0.23	<0.001
BUN (mmol/L)	4.70 (4.00-5.60)	4.65 (4.00-5.60)	4.80 (3.90-5.50)	0.02	0.688
UA (ummol/L)	343.80 (307.40-376.90)	337.55 (303.20-372.40)	356.80 (317.40-390.20)	0.25	<0.001
TC (mmol/L)	5.14 ± 0.85	5.07 ± 0.82	5.27 ± 0.91	0.23	<0.001
LDL-c (mmol/L)	3.18 ± 0.83	3.20 ± 0.79	3.12 ± 0.92	0.10	0.031
HDL-c (mmol/L)	1.12 ± 0.22	1.15 ± 0.22	1.05 ± 0.19	0.52	<0.001
TyG index	9.00 (8.76-9.21)	8.87 (8.66-9.00)	9.32 (9.22-9.42)	2.79	<0.001
2hPG (mmol/L)	7.49 ± 1.46	7.38 ± 1.31	7.72 ± 1.69	0.23	<0.001
Homocysteine (umol/L)	14.56 (14.10-15.06)	13.06 (12.06-15.06)	14.06 (12.40-15.06)	0.04	<0.001
ALT (U/L)	24.00 (17.00-35.00)	24.00 (17.00-35.00)	23.00 (17.00-34.00)	0.03	0.468
AST (U/L)	23.00 (19.00-28.00)	23.00 (19.00-28.00)	22.00 (19.00-27.00)	0.04	0.337
WBC (10^9/L)	6.46 (5.86-6.56)	6.46 (5.85-6.56)	6.46 (5.87-6.53)	0.06	0.328
RBC (10^9/L)	4.96 (4.83-5.08)	4.96 (4.86-5.10)	4.94 (4.82-5.12)	0.08	0.071
Hemoglobin (g/L)	150.00 (147.00-155.00)	150.00 (147.75-155.00)	150.00 (146.00-153.00)	0.06	0.240
RDW (%)	12.88 (12.60-12.90)	12.88 (12.60-12.90)	12.88 (12.50-12.88)	0.03	0.520
Platelets (10^9/L)	237.00 (218.00-245.00)	237.00 (217.00-239.25)	237.00 (225.00-254.00)	0.13	0.001
PRA (pg/ml)	2.95 (1.57-3.38)	2.84 (1.59-3.38)	3.30 (1.54-3.80)	0.11	0.048
Aldosterone (ng/ml)	16 (13–18)	16 (13–18)	17 (14–19)	0.04	0.312
OSBP (mmHg)	152.47 ± 11.92	151.75 ± 11.34	153.98 ± 12.92	0.18	<0.001
ODBP (mmHg)	99.93 ± 8.81	99.33 ± 8.22	101.17 ± 9.82	0.20	<0.001
OPP (mmHg)	52.55 ± 10.93	52.42 ± 11.25	52.80 ± 10.26	0.04	0.460
HR (beats/min)	78.48 ± 9.39	77.93 ± 9.52	79.62 ± 9.02	0.18	<0.001
24hSBP (mmHg)	135.68 ± 10.99	135.04 ± 10.57	136.99 ± 11.71	0.17	<0.001
24hDBP (mmHg)	87.80 ± 8.80	87.15 ± 8.40	89.15 ± 9.44	0.22	<0.001
24hPP (mmHg)	47.88 ± 8.93	47.89 ± 9.21	47.84 ± 8.34	0.01	0.897
DSBP (mmHg)	140.12 ± 11.04	139.55 (10.60)	141.30 (11.82)	0.16	<0.001
DDBP (mmHg)	91.26 ± 9.02	90.58 ± 8.69	92.65 ± 9.51	0.23	<0.001
DPP (mmHg)	48.86 ± 9.11	48.96 ± 9.27	48.65 ± 8.76	0.03	0.470
NSBP (mmHg)	126.86 ± 12.57	125.98 ± 12.14	128.67 ± 13.25	0.21	<0.001
NDBP (mmHg)	80.75 ± 9.84	80.01 ± 9.24	82.30 ± 10.84	0.23	<0.001
NPP (mmHg)	46.10 ± 9.00	45.97 ± 9.21	46.37 ± 8.57	0.04	0.349

Data are presented as means ± SDs, medians (interquartile ranges), or n (%). BMI, body mass index; eGFR, estimated glomerular filtration rate; UACR, urine albumin-creatinine ratio; BUN, blood urea nitrogen; UA, Uric acid; TC, total cholesterol; LDL-c, low density lipoprotein cholesterol; HDL-c, high density lipoprotein cholesterol; 2hPG, 2-hour postprandial blood glucose; ALT, alanine aminotransferase; AST, aspertate aminotransferase; WBC, white blood cell; RBC, red blood cell; RDW, red cell distribution width; PRA, plasma renin activity; OSBP, office systolic blood pressure; ODBP, office diastolic blood pressure; OPP, office pulse pressure; HR, heart rate; 24hSBP, 24-hour systolic blood pressure;24hDBP, 24-hour diastolic blood pressure;24hPP, 24-hour pulse pressure; DSBP, daytime systolic blood pressure; DDBP, daytime diastolic blood pressure; DPP, daytime pulse pressure; NSBP, night-time systolic blood pressure; NDBP, night-time diastolic blood pressure; NPP, night-time pulse pressure.

The PSM-matched population showed differences in the proportions of BMI, eGFR, creatinine, UACR, UA, HDL-C, 2hPG, RBC, platelets, PRA, and DPP (all *P* < 0.05) (**Supplementary Table 1**).

### 3.2 Analysis of the association between the TyG index and the incidence of MAU

**Figure 2:**
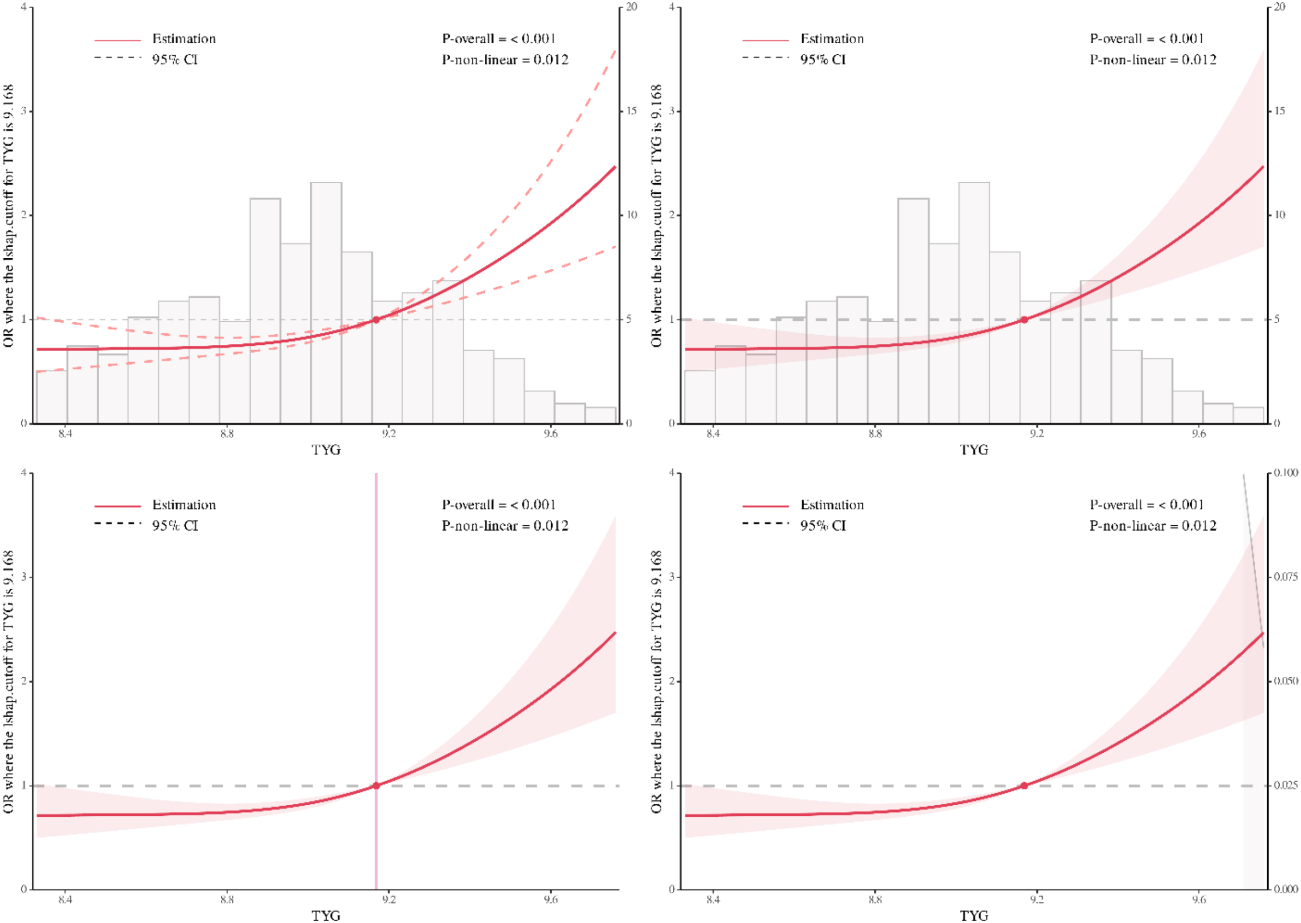
The TyG index exhibits a nonlinear relationship with the risk of MAU (*P*-overall<0.001, *P*-non-linear=0.012).

The analysis of the RCS curve revealed a positive dose-response relationship between the TyG index and MAU, indicating that the incidence of MAU rises with increasing TyG index levels (**Figure 2**; *P* for overall<0.001). In **Supplementary Figure 1**, we also discovered non-linear associations between the TyG index and MAU after PSM (*P* for overall<0.001).

### 3.3 Association between TyG index and MAU in essential hypertension patients in the PS-matched cohort

As shown in **Supplementary Table 2**, the results of a univariate analysis are unambiguous. **Table 2** shows the estimated odds ratios (ORs) and 95% confidence intervals (CIs) for the TyG index in relation to MAU after PSM. As a categorical variable, the prevalence of MAU increased remarkably among patients in the high TyG index group compared to that observed in the low TyG index group (crude model: OR 1.85, 95% CI 1.43–2.40, *P* < 0.0001; Model I: OR 2.41, 95% CI 1.79–3.26, *P* < 0.0001; Model II: OR 2.37, 95% CI 1.73–3.26, *P* < 0.0001). As a successive variable, after adjusting for the clinical confounders listed, an SD increase in TyG index level was related to a 64% increased risk of in-hospital mortality (OR 1.64, 95% CI 1.39–1.95, *P* < 0.0001) in the fully adjusted model. When the TyG index was assessed in the three models, patients with a high TyG index also had a higher risk of MAU (OR 3.33, 95%CI 2.21–5.04, *P* < 0.0001) than that observed in patients in the low TyG index group in the adjusted model.

**Table 2:** Adjusted odds ratios for the incidence of MAU according to the presence of high TyG index in the PS-matched cohort.

Exposure	Non-adjusted		Adjust I		Adjust II	
	OR (95%CI)	*P*-value	OR (95%CI)	*P*-value	OR (95%CI)	*P*-value
TyG index as a continuous variable	2.96 (1.82, 4.82)	<0.0001	4.85 (2.70, 8.73)	<0.0001	6.22 (3.31, 11.68)	<0.0001
TyG index per SD increase	1.34 (1.18, 1.53)	<0.0001	1.54 (1.31, 1.80)	<0.0001	1.64 (1.39, 1.95)	<0.0001
TyG index group						
low	1.0		1.0		1.0	
high	1.85 (1.43, 2.40)	<0.0001	2.41 (1.79, 3.26)	<0.0001	2.37 (1.73, 3.26)	<0.0001
TyG index tertile						
Tertile1	1.0		1.0		1.0	
Tertile2	0.82 (0.59, 1.13)	0.2200	1.07 (0.74, 1.56)	0.7137	1.13 (0.75, 1.70)	0.5496
Tertile3	1.83 (1.33, 2.52)	0.0002	3.03 (2.06, 4.43)	<0.0001	3.33 (2.21, 5.04)	<0.0001
*P* for trend	1.37 (1.17, 1.61)	0.0001	1.77 (1.46, 2.15)	<0.0001	1.87 (1.52, 2.31)	<0.0001

OR (95%CI) *P* value. Non-adjusted model adjusted for: None. Adjust I model adjusted for: BMI, age, smoking, eGFR, and gender. Adjust II model adjusted for: BMI, age, smoking, eGFR, gender, OSBP, ODBP, HR, TC, PRA, and aldosterone.

### 3.4 Sensitivity analysis

According to these findings, a sensitivity analysis was applied to further confirm the association between the TyG index and the incidence of MAU in the two cohorts examined, and a weighted cohort was developed with the estimated PS through the development of an IPTW model (**Table 3**). We also used non-adjusted, partially adjusted, and fully adjusted models across the two cohorts. In the original cohort, a high risk of MAU was noted in cases with a high TyG index (crude model: OR 1.78, 95% CI 1.48–2.15, *P* < 0.001; Model I: OR 2.24, 95% CI 1.80–2.79, *P* < 0.001; Model II: OR 2.06, 95% CI 1.64–2.59, *P* < 0.001) after adjusting for different covariates. The results remained marked in the weighted cohort (crude model: OR 1.78, 95% CI 1.57–2.02, *P* < 0.001; Model I: OR 2.03, 95% CI: 1.76–2.35, *P* < 0.001; Model II: OR 2.16, 95% CI 1.85–2.52, *P* < 0.001). The results were also significant when the TyG index was used as a successive variable. When the TyG index was assessed in the three models, patients with a high TyG index also had a higher risk of MAU than that observed in patients with a low TyG index in both original and weighted cohorts.

**Table 3:** Association of TyG and MAU among essential hypertension in the original (A) and weighted cohorts (B).

Exposure(A)	Non-adjusted		Adjust I		Adjust II	
	OR (95%CI)	*P*-value	OR (95%CI)	*P*-value	OR (95%CI)	*P*-value
TyG index as a continuous variable	2.25 (1.69, 3.00)	<0.0001	3.19 (2.26, 4.51)	<0.0001	3.22 (2.22, 4.67)	<0.0001
TyG index per SD increase	1.29 (1.18, 1.41)	<0.0001	1.44 (1.29, 1.60)	<0.0001	1.44 (1.28, 1.62)	<0.0001
TyG index group		<0.0001		<0.0001		<0.0001
low	1.0		1.0		1.0	
high	1.78 (1.48, 2.15)	<0.0001	2.24 (1.80, 2.79)	<0.0001	2.06 (1.64, 2.59)	<0.0001
TyG index tertile						
Tertile1	1.0		1.0		1.0	
Tertile2	1.42 (1.14, 1.77)	0.0018	1.64 (1.28, 2.11)	<0.0001	1.73 (1.33, 2.25)	<0.0001
Tertile3	2.04 (1.64, 2.53)	<0.0001	2.66 (2.06, 3.44)	<0.0001	2.54 (1.93, 3.34)	<0.0001
*P* for trend	1.43 (1.28, 1.59)	<0.0001	1.63 (1.44, 1.85)	<0.0001	1.59 (1.39, 1.82)	<0.0001

Exposure(B)	Non-adjusted		Adjust I		Adjust II	
	OR (95%CI)	*P*-value	OR (95%CI)	*P*-value	OR (95%CI)	*P*-value
TyG index as a continuous variable	2.34 (1.86, 2.95)	<0.0001	3.69 (2.81, 4.84)	<0.0001	4.87 (3.62, 6.54)	<0.0001
TyG index per SD increase	1.30 (1.21, 1.40)	<0.0001	1.50 (1.38, 1.64)	<0.0001	1.64 (1.50, 1.80)	<0.0001
TyG index group						
low	1.0		1.0		1.0	
high	1.78 (1.57, 2.02)	<0.0001	2.03 (1.76, 2.35)	<0.0001	2.16 (1.85, 2.52)	<0.0001
TyG index tertile						
Tertile1	1.0		1.0		1.0	
Tertile2	1.16 (0.96, 1.39)	0.1273	1.57 (1.26, 1.94)	<0.0001	1.70 (1.35, 2.13)	<0.0001
Tertile3	1.77 (1.50, 2.09)	<0.0001	2.34 (1.93, 2.83)	<0.0001	2.69 (2.18, 3.31)	<0.0001
*P* for trend	1.36 (1.25, 1.47)	<0.0001	1.52 (1.39, 1.67)	<0.0001	1.63 (1.47, 1.80)	<0.0001

OR (95%CI) *P* value. Non-adjusted model adjusted for: None. Adjust I model adjusted for: BMI, age, smoking, eGFR, and gender. Adjust II model adjusted for: BMI, age, smoking; eGFR, gender, OSBP, ODBP, HR, TC, PRA, and aldosterone.

### 3.5 E-value analyses

**Figure 3:**
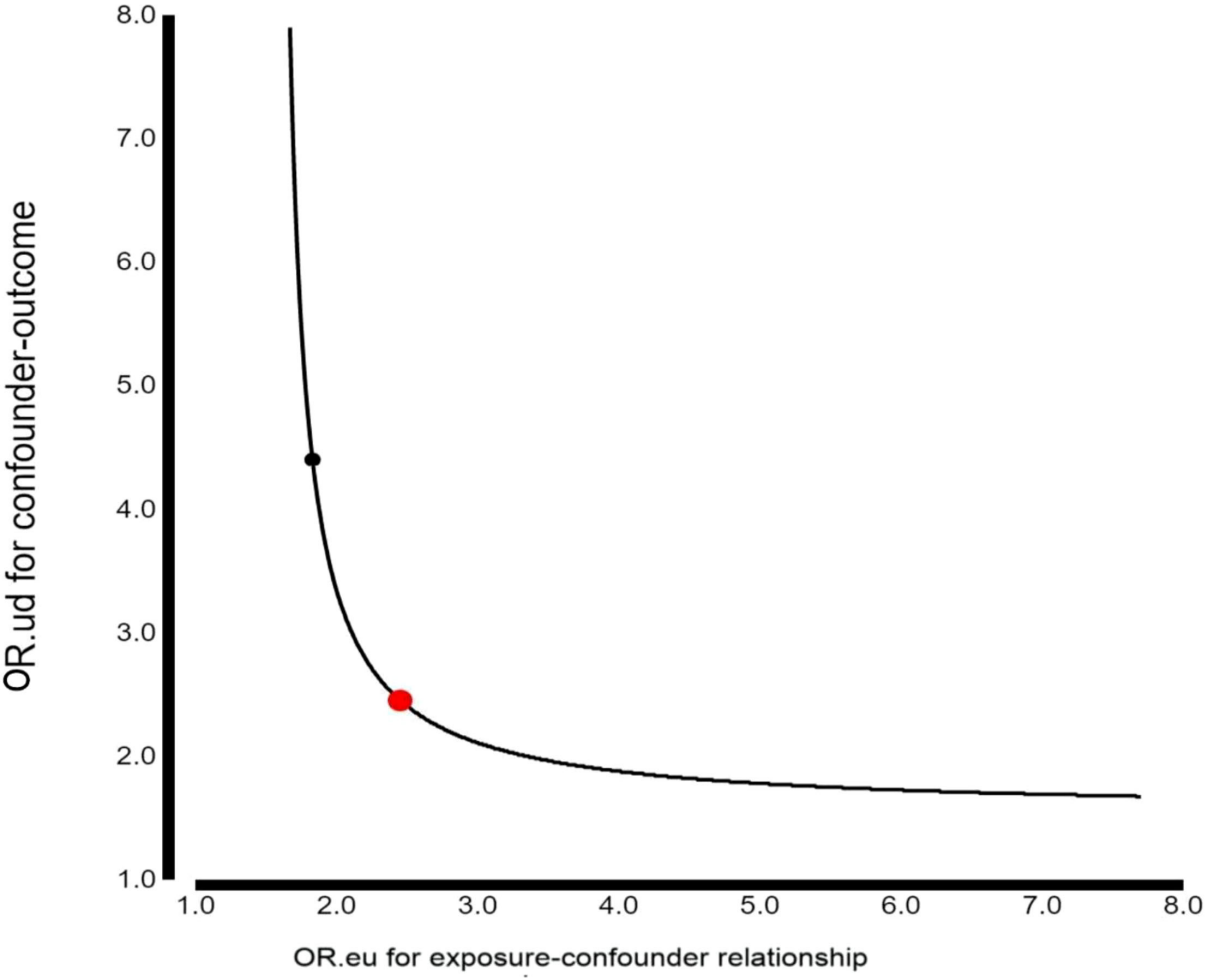
E-value sensitivity analysis. An unmeasured confounder would require an odds ratio of at least 2.45 with both the TyG index and MAU to fully explain the observed association (adjusted OR 2.37, 95% CI 1.73-3.26).

Using Van der Weele and Ding’s E-value methodology, we conducted a sensitivity analysis to identify possible confounding effects (26). In the current study, the adjusted OR for the associations between TyG index and MAU was 2.37 (95% CI 1.73–3.26). An unmeasured confounder could fully account for the association of intensification with MAU if it were associated with the exposure and outcome with an OR of 2.45 (lower confidence limit 1.85; higher confidence limit 4.21) (**Figure 3**). Considering this E-value, fewer confounding factors seemed to affect the current association between the TyG index and MAU.

### 3.6 Subgroup analysis

**Figure 4:**
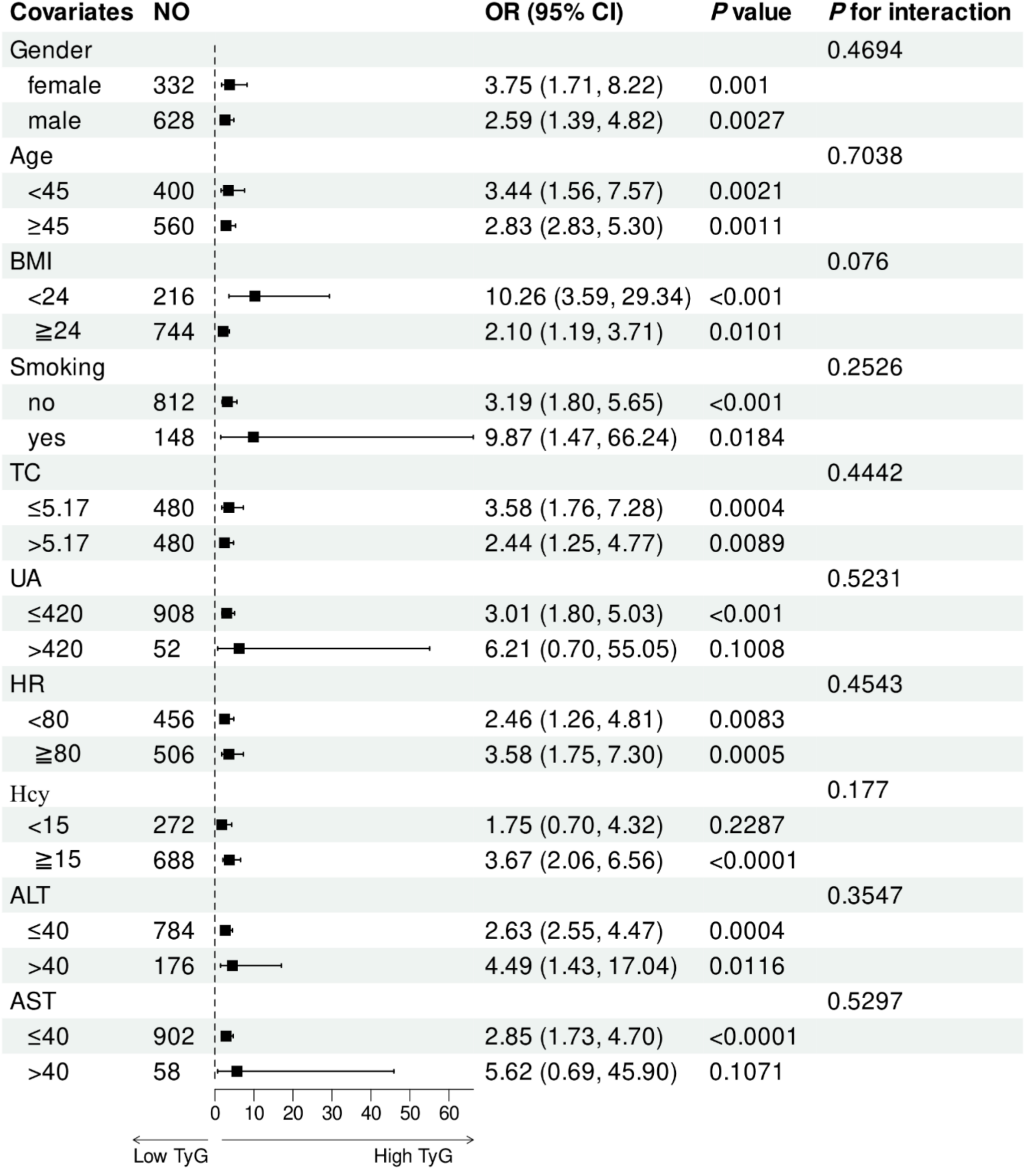
Subgroup analyses showed that a high TyG index is consistently associated with increased MAU risk across all subgroups, with no significant interactions detected (all *P* for interaction > 0.05).

We performed stratified analysis to evaluate the robustness of our findings in diverse subgroups following PSM. After adjusting for potential confounders, we found no interactions between gender, age, BMI, smoking, TC, UA, HR, Hcy, ALT, and AST (**Table 4**; **Figure 4**). The high TyG index (≥9.125) group had a higher MAU rate than that in the low TyG index (<9.125) group for all subgroups. However, whether the TyG index interacted with any of the subgroup factors (*P* > 0.05) was not evident when we analyzed the interaction between the TyG index and each factor.

**Table 4:** Stratified analysis for the effect of each covariate.

Covariates	No	OR (95%CI)	Sub-group *P* value	*P* for interaction
Gender				0.4694
female	332	3.75 (1.71, 8.22)	0.0010	
male	628	2.59 (1.39, 4.82)	0.0027	
Age, years				0.7038
<45	400	3.44 (1.56, 7.57)	0.0021	
≧45	560	2.83 (1.51, 5.30)	0.0011	
BMI, kg/m2				0.0760
<24	216	10.26 (3.59, 29.34)	<0.001	
≧24	744	2.10 (1.19, 3.71)	0.0101	
Smoking				0.2526
no	812	3.19(1.80, 5.65)	<0.001	
yes	148	9.87(1.47, 66.24)	0.0184	
TC,mmol/L				0.4442
≤5.17	480	3.58(1.76, 7.28)	0.0004	
>5.17	480	2.44(1.25, 4.77)	0.0089	
UA,umol/L				0.5231
≤420	908	3.01 (1.80, 5.03)	<0.001	
>420	52	6.21(0.70, 55.05)	0.1008	
HR, beats/min				0.4543
<80	456	2.46(1.26, 4.81)	0.0083	
≧80	506	3.58(1.75, 7.30)	0.0005	
Hcy,mmol/L				0.1770
<15	272	1.75(0.70, 4.32)	0.2287	
≧15	688	3.67 (2.06, 6.56)	<0.0001	
ALT, U/L				0.3547
≤40	784	2.63 (1.55, 4.47)	0.0004	
>40	176	4.94 (1.43, 17.04)	0.0116	
AST, U/L				0.5297
≤40	902	2.85 (1.73, 4.70)	<0.0001	
>40	58	5.62(0.69, 45.90)	0.1071	

### 3.7 Prediction of MAU

To evaluate the predictive value of the TyG index for MAU among individuals with essential hypertension, the non-parametric bootstrap method (500 resampling iterations) was employed to calculate the AUC and its 95% CI (**Supplementary Figure 2**). Analysis of individual predictors revealed that the TyG index alone had limited predictive performance (AUC 0.572, 95% CI 0.545-0.593. At the optimal TyG index threshold of 9.125, the specificity was 72.8%, and the sensitivity was 39.9%, indicating that the TyG index alone lacks sufficient discriminative power for clinical prediction. To enhance predictive performance, models were developed with specific adjustments for BMI, age, gender, UA, creatinine, OSBP, ODBP, and HR, using key variables identified through LASSO and stepwise regression analysis. The prediction model achieved an AUC of 0.723 (95% CI 0.701-0.745) (**Supplementary Figure 3**). At the optimal threshold of−0.461, specificity, sensitivity, and negative predictive value were 62.7%, 70.2%, and 47.6%, respectively.

## 4 Discussion

The principal finding of this study is that a higher TyG index is independently associated with an increased risk of MAU in newly diagnosed, treatment-naïve patients with essential hypertension (adjusted OR 2.37, 95% CI 1.73-3.26). This association remained robust across multiple sensitivity analyses employing PSM and inverse IPTW and exhibited a nonlinear dose–response relationship. The novelty of our study lies in systematically establishing this link specifically within a “clean” cohort of incident hypertension, rigorously excluding diabetes, prior antihypertensive treatment, and established chronic kidney disease. This emphasizes the potential value of assessing metabolic renal risk at the very outset of hypertension management.

These findings align with previous research exploring the relationship between the TyG index and MAU. Several population-based studies have demonstrated that the TyG index is associated with a higher risk of nephric microvascular damage, even after adjustment for traditional cardiovascular risk factors (27, 28). In hypertensive cohorts, it was reported that there was a positive correlation between the TyG index and albuminuria (29). A previous study incorporating renal damage indicators reported that a higher TyG index was associated with microalbuminuria (MAU) and DKD (30). However, that study included patients who had previously been diagnosed with hypertension, and the vast majority were taking antihypertensive therapy. Such confounding factors may affect the levels of metabolic abnormalities, including IR (31), resulting in lower or higher diagnostic performances of the TyG index. Given that newly diagnosed hypertensive patients have not received any treatment, they may be more likely to have impaired glycolipid metabolism and higher blood pressure (32). Therefore, we hypothesize that the diagnostic performance of the TyG index for MAU may differ between newly diagnosed and previously diagnosed hypertensive patients.

The association observed between the TyG index and MAU provides further clinical epidemiological support for the pivotal role of IR in the early phase of hypertensive renal injury. As a surrogate marker of IR, an elevated TyG index may promote MAU through multiple interrelated pathways:1) Endothelial dysfunction and micro-inflammation: The lipotoxic state and chronic low-grade inflammation associated with IR can impair glomerular endothelial function and increase vascular permeability (6, 33). 2) Oxidative stress: Elevated free fatty acids and dysglycemia increase reactive oxygen species production, leading to direct podocyte and tubular epithelial injury (34, 35). 3) Altered glomerular hemodynamics: IR is often accompanied by compensatory hyperinsulinemia and activation of the RAAS, contributing to increased intraglomerular pressure (4, 36, 37). These mechanisms align with the trend observed in our subgroup analyses, where higher TyG index and blood pressure levels appeared synergistic. Our findings are consistent with previous reports linking the TyG index to albuminuria in broader populations (38–43), but they extend this association to an earlier stage in the disease continuum, thereby identifying a critical window for intervention.

Furthermore, our research clearly indicates that there is a significant non-linear relationship between the TyG index and MAU, particularly among patients with a higher TyG index, where the incidence of MAU is notably increased. This phenomenon is consistent with the known pathophysiological mechanisms of diabetic nephropathy, including the synergistic effects of multiple mechanisms such as glomerular hypertension, activation of inflammatory responses, and oxidative stress, which can accelerate renal microvascular damage and thereby promote the occurrence and development of proteinuria (33, 34, 43–45). Therefore, introducing TyG index screening into the routine health management of hypertensive patients not only helps in the early detection of kidney damage but also provides an effective basis for stratified management and individualized intervention.

To verify the robustness of the main results, this study employed a sensitivity analysis based on propensity score weighting to further control for potential confounding factors. Sensitivity analysis using IPTW to construct a weighted cohort revealed that the risk of microalbuminuria in the high TyG group remained significantly elevated (OR 2.16, 95% CI 1.85–2.52). This effect size was similar to the result of the original cohort analysis (OR 2.06, 95% CI 1.64–2.59) and consistent with previous studies on the association between the TyG index and albuminuria (39). This indicates that the association between the TyG index and the risk of MAU remains robust after different statistical methods and adequate control of confounding factors, providing strong support for the research conclusion. This finding further emphasizes the value of the TyG index in the risk assessment of early renal injury in hypertensive patients. A recent meta-analysis of longitudinal cohort studies confirmed that a higher baseline TyG index was significantly associated with an increased risk of incident CKD (46). This is not only consistent with the results of previous literature but also suggests that in clinical practice, the dynamic assessment of the TyG index should be emphasized, and it should be used as an economical, effective, and non-invasive marker for identifying early cardiovascular abnormalities and stratifying metabolic risks (6, 47). By monitoring the dynamic changes of the TyG index, valuable guidance can be provided for disease management, thereby achieving early detection and improved prognosis.

Although the discriminatory power of the TyG index alone for MAU is modest (AUC 0.58), this does not diminish its potential utility as a simple, economical, and accessible risk-stratification tool. In clinical practice, particularly in primary care or resource-limited settings, the TyG index, calculable from routine fasting blood tests, can efficiently identify high-risk patients who should be prioritized for confirmatory, albeit more costly, urinary albumin-to-creatinine ratio testing. For patients with an elevated TyG index, clinicians could initiate lifestyle modifications more proactively and consider antihypertensive agents with favorable metabolic profiles, such as RAAS inhibitors.

This study’s findings offer valuable insights into the connection between the TyG index and MAU in hypertensive patients, though several limitations should be noted. First, the study population was limited to newly diagnosed hypertensive patients from a single center, which may restrict the generalizability of the results to different populations or medical settings. Therefore, future studies should be conducted in a multi-center and diverse setting for further validation to enhance the external applicability of the research. Second, although we used non-insulin-based indicators to assess IR, we did not employ the gold standard methods for directly measuring insulin sensitivity, such as the hyperinsulinemic-euglycemic clamp technique. These direct measurement methods are not easily implemented in large observational studies due to their high cost and invasiveness. Third, as the study was retrospective in design, important information that could affect the association between the TyG index and MAU, such as detailed lifestyle factors (dietary patterns, exercise habits), medication adherence, and family history of metabolic diseases, was not recorded. This limits the ability to conduct a more in-depth analysis of the observed associations. Fourth, although we conducted extensive subgroup analyses, the possibility of residual confounding or unmeasured variables still exists, which may affect the robustness of the conclusions. Therefore, future studies should be conducted in larger and more diverse populations, integrating longitudinal data on potential confounding factors, to validate and expand upon our research findings. Finally, the discriminative ability of the TyG index alone for MAU, as indicated by the AUC, is modest. Its clinical utility may be greater when integrated into a multifactorial risk assessment rather than as a standalone diagnostic tool. Future research should focus on: 1) developing integrated risk prediction models that combine the TyG index with blood pressure, renal function markers, and other clinical parameters; 2) conducting prospective cohort studies to examine whether reducing the TyG index (via lifestyle or pharmacological intervention) can prevent the development or progression of MAU; and 3) exploring the value of the TyG index in guiding personalized treatment decisions in hypertension.

## 5 Conclusions

In conclusion, a higher TyG index is independently and robustly associated with an increased risk of MAU in newly diagnosed patients with essential hypertension. While its standalone discriminatory ability is modest, the TyG index serves as an easily accessible marker of metabolic dysregulation. Its incorporation into the initial assessment of hypertension may help identify individuals at heightened risk for early renal injury, thereby informing timely and targeted management strategies aimed at improving renal and cardiovascular outcomes.

## Data Availability

The original contributions presented in the study are included in the article/**Supplementary Material**. Further inquiries can be directed to the corresponding author.
