# Supplementary material for: Propensity score analysis of triglyceride-glucose index in newly diagnosed patients with essential hypertension as a predictor of microalbuminuria
Source: Front Endocrinol (Lausanne). 2026 Mar 9;17:1737230. doi: 10.3389/fendo.2026.1737230 (PMC13006190; doi:10.3389/fendo.2026.1737230)
Supplement: Supplementary file 1 [file DataSheet1.docx]

Table S1 Baseline characteristics of participants according to TyG index group in the PS-matched cohort

| TyG index group | Total | Low TyG index | High TyG index | SMD | *P*-value |
| --- | --- | --- | --- | --- | --- |
| N | 960 | 480 | 480 |  |  |
| BMI,kg/m^2^ | 26.12 (24.20-28.54) | 25.72 (23.88-28.54) | 26.45 (24.85-28.45) | 0.06 | 0.017 |
| Age,years | 47.17±7.52 | 47.46±7.07 | 46.88±7.94 | 0.08 | 0.237 |
| Gender |  |  |  | 0.02 | 0.786 |
| female | 332 (34.58%) | 168 (35.00%) | 164 (34.17%) |  |  |
| male | 628 (65.42%) | 312 (65.00%) | 316 (65.83%) |  |  |
| Smoking |  |  |  | 0.02 | 0.721 |
| no | 812 (84.58%) | 404 (84.17%) | 408 (85.00%) |  |  |
| yes | 148 (15.42%) | 76 (15.83%) | 72 (15.00%) |  |  |
| eGFR,ml/min/1.73m^2^ | 104.29 (92.77-117.91) | 100.98 (90.13-114.89) | 108.10 (95.93-122.72) | 0.18 | <0.001 |
| Creatinine,umol/L | 70.00 (59.00-79.00) | 72.00 (59.75-81.00) | 68.50 (59.00-78.00) | 0.16 | 0.008 |
| UACR,mg/g | 27.30 (11.90-43.60) | 26.30 (9.73-36.47) | 31.15 (12.30-48.78) | 0.14 | 0.002 |
| BUN,,mmol/L | 4.90 (4.10-5.60) | 4.70 (4.20-5.60) | 4.95 (4.00-5.60) | 0.05 | 0.395 |
| Uric acid,ummol/L | 352.10 (310.52-387.07) | 346.35 (303.28-383.30) | 359.00 (313.60-391.35) | 0.13 | 0.037 |
| TC,mmol/L | 5.19±0.89 | 5.19±0.89 | 5.20±0.89 | 0.00 | 0.945 |
| LDL,mmol/L | 3.13±0.88 | 3.14±0.84 | 3.12±0.91 | 0.02 | 0.716 |
| HDL,mmol/L | 1.13±0.24 | 1.27±0.22 | 1.00±0.17 | 1.36 | <0.001 |
| TyG index | 9.12 (8.95-9.28) | 8.95 (8.79-9.06) | 9.28 (9.20-9.40) | 2.45 | <0.001 |
| 2hPG,mmol/L | 7.57±1.69 | 7.35±1.47 | 7.78±1.87 | 0.26 | <0.001 |
| Homocysteine,umol/L | 15.06 (13.78-15.06) | 15.06 (14.05-15.06) | 15.06 (13.10-15.06) | 0.09 | 0.060 |
| ALT,U/L | 23.00 (16.00-34.00) | 23.00 (16.00-34.00) | 24.00 (16.00-35.00) | 0.02 | 0.624 |
| AST,U/L | 22.00 (19.00-27.25) | 22.00 (19.00-27.25) | 22.00 (19.00-27.25) | 0.09 | 0.633 |
| WBC,10^9/L | 6.46 (5.85-6.47) | 6.46 (5.82-6.46) | 6.46 (5.87-6.56) | 0.08 | 0.496 |
| RBC,10^9/L | 4.96 (4.86-5.08) | 4.96 (4.96-5.13) | 4.96 (4.80-5.00) | 0.13 | <0.001 |
| Hemoglobin,g/L | 150.00 (148.00-155.00) | 150.00 (150.00-157.00) | 150.00 (147.00-153.00) | 0.11 | 0.079 |
| RDW,% | 12.88 (12.60-12.88) | 12.88 (12.60-12.88) | 12.88 (12.57-12.88) | 0.01 | 0.907 |
| Platelets,10^9/L | 237.00 (219.75-248.25) | 237.00 (219.00-240.00) | 237.00 (222.75-253.00) | 0.09 | 0.031 |
| PRA,pg/ml | 2.99 (1.59-3.38) | 2.84 (1.59-3.38) | 3.30 (1.59-3.89) | 0.21 | 0.043 |
| Aldosterone,ng/ml | 16 (13-17) | 16 (13-17) | 16 (13-17) | 0.00 | 0.954 |
| OSBP,mmHg | 152.67±12.29 | 152.08±11.95 | 153.25±12.61 | 0.09 | 0.141 |
| ODBP,mmHg | 99.82±9.06 | 99.55±8.40 | 100.09±9.68 | 0.06 | 0.355 |
| OPP,mmHg | 52.85±10.74 | 52.53±11.15 | 53.16±10.30 | 0.06 | 0.367 |
| HR,beats/min | 79.10±9.55 | 78.72±9.84 | 79.48±9.25 | 0.08 | 0.219 |
| 24hSBP,mmHg | 136.42±11.47 | 136.62±11.63 | 136.22±11.31 | 0.04 | 0.581 |
| 24hDBP,mmHg | 88.12±9.22 | 87.84±9.36 | 88.39±9.07 | 0.06 | 0.356 |
| 24hPP,mmHg | 48.30±8.95 | 48.78±9.50 | 47.83±8.36 | 0.11 | 0.097 |
| DSBP,mmHg | 140.72±11.65 | 140.97±11.66 | 140.47±11.65 | 0.04 | 0.507 |
| DDBP,mmHg | 91.62±9.49 | 91.28±9.64 | 91.97±9.33 | 0.07 | 0.259 |
| DPP,mmHg | 49.10±9.27 | 49.69±9.67 | 48.50±8.83 | 0.13 | 0.046 |
| NSBP,mmHg | 127.67±12.91 | 127.51±13.14 | 127.83±12.69 | 0.03 | 0.697 |
| NDBP,mmHg | 81.18±10.15 | 80.79±10.12 | 81.57±10.17 | 0.08 | 0.237 |
| NPP,mmHg | 46.49±9.13 | 46.72±9.51 | 46.27±8.74 | 0.05 | 0.446 |

Data are presented as means±SDs, medians (interquartile ranges), or n (%).

Notes: BMI, body mass index; eGFR, estimated glomerular filtration rate; UACR, urine albumin-creatinine ratio; BUN, blood urea nitrogen; UA,Uric acid; TC, total cholesterol; LDL-c, low density lipoprotein cholesterol; HDL-c, high density lipoprotein cholesterol; 2hPG, 2-hour postprandial blood glucose; ALT, alanine aminotransferase; AST, aspertate aminotransferase; WBC, white blood cell; RBC, red blood cell; RDW, red cell distribution width; PRA, plasma renin activity; OSBP, office systolic blood pressure;ODBP, office diastolic blood pressure;OPP, office pulse pressure;HR, heart rate; 24hSBP, 24-hour systolic blood pressure;24hDBP, 24-hour diastolic blood pressure;24hPP, 24-hour pulse pressure; DSBP,daytime systolic blood pressure;DDBP,daytime diastolic blood pressure;DPP, daytime pulse pressure; NSBP, night-time systolic blood pressure;NDBP,night-time diastolic blood pressure;NPP,night-time pulse pressure.

Table S2 Influencing factors for TyG index in the PS-matched population by univariate logistic-regression analysis.

| Variable Name | MAU[OR(95%CI)] | *P* value |
| --- | --- | --- |
| BMI | 0.97 (0.95, 1.00) | 0.0204 |
| Age | 1.03 (1.02, 1.04) | <0.0001 |
| Gender |  | 0.0157 |
| female | 1.0 |  |
| male | 0.80 (0.67, 0.96) |  |
| Smoking |  | <0.0001 |
| NO | 1.0 |  |
| Yes | 12.37 (8.75, 17.51) |  |
| eGFR | 0.99 (0.99, 1.00) | <0.0001 |
| Cr | 1.00 (0.99, 1.01) | 0.8655 |
| BUN | 1.05 (0.97, 1.13) | 0.2651 |
| UA | 1.01 (1.01, 1.01) | <0.0001 |
| CHO | 1.27 (1.15, 1.41) | <0.0001 |
| LDL | 1.28 (1.15, 1.43) | <0.0001 |
| HDL | 0.34 (0.22, 0.51) | <0.0001 |
| TYG | 2.25 (1.69, 3.00) | <0.0001 |
| HPG | 1.03 (0.97, 1.10) | 0.2671 |
| HCY | 0.99 (0.98, 1.01) | 0.2453 |
| ALT | 1.00 (0.99, 1.00) | 0.4080 |
| AST | 1.00 (0.99, 1.01) | 0.8723 |
| WBC | 1.00 (0.94, 1.07) | 0.8776 |
| RBC | 0.70 (0.55, 0.90) | 0.0046 |
| HB | 0.99 (0.98, 1.00) | 0.0138 |
| RDW | 1.13 (1.00, 1.29) | 0.0552 |
| PLT | 1.00 (1.00, 1.00) | 0.8196 |
| CSBP | 1.04 (1.04, 1.05) | <0.0001 |
| CDBP | 1.05 (1.04, 1.06) | <0.0001 |
| CPP | 1.02 (1.01, 1.03) | <0.0001 |
| HR | 1.01 (1.00, 1.02) | 0.0806 |
| 24hSBP | 1.06 (1.05, 1.07) | <0.0001 |
| 24hDBP | 1.07 (1.06, 1.08) | <0.0001 |
| 24hPP | 1.01 (1.00, 1.02) | 0.0035 |
| DSBP | 1.03 (1.02, 1.03) | <0.0001 |
| DDBP | 1.04 (1.03, 1.05) | <0.0001 |
| DPP | 1.00 (0.99, 1.01) | 0.9870 |
| NSBP | 1.11 (1.10, 1.12) | <0.0001 |
| NDBP | 1.12 (1.11, 1.14) | <0.0001 |
| NPP | 1.04 (1.03, 1.05) | <0.0001 |
| PRA | 1.03 (1.01, 1.06) | 0.0145 |
| ALD | 0.79 (0.10, 6.04) | 0.8175 |


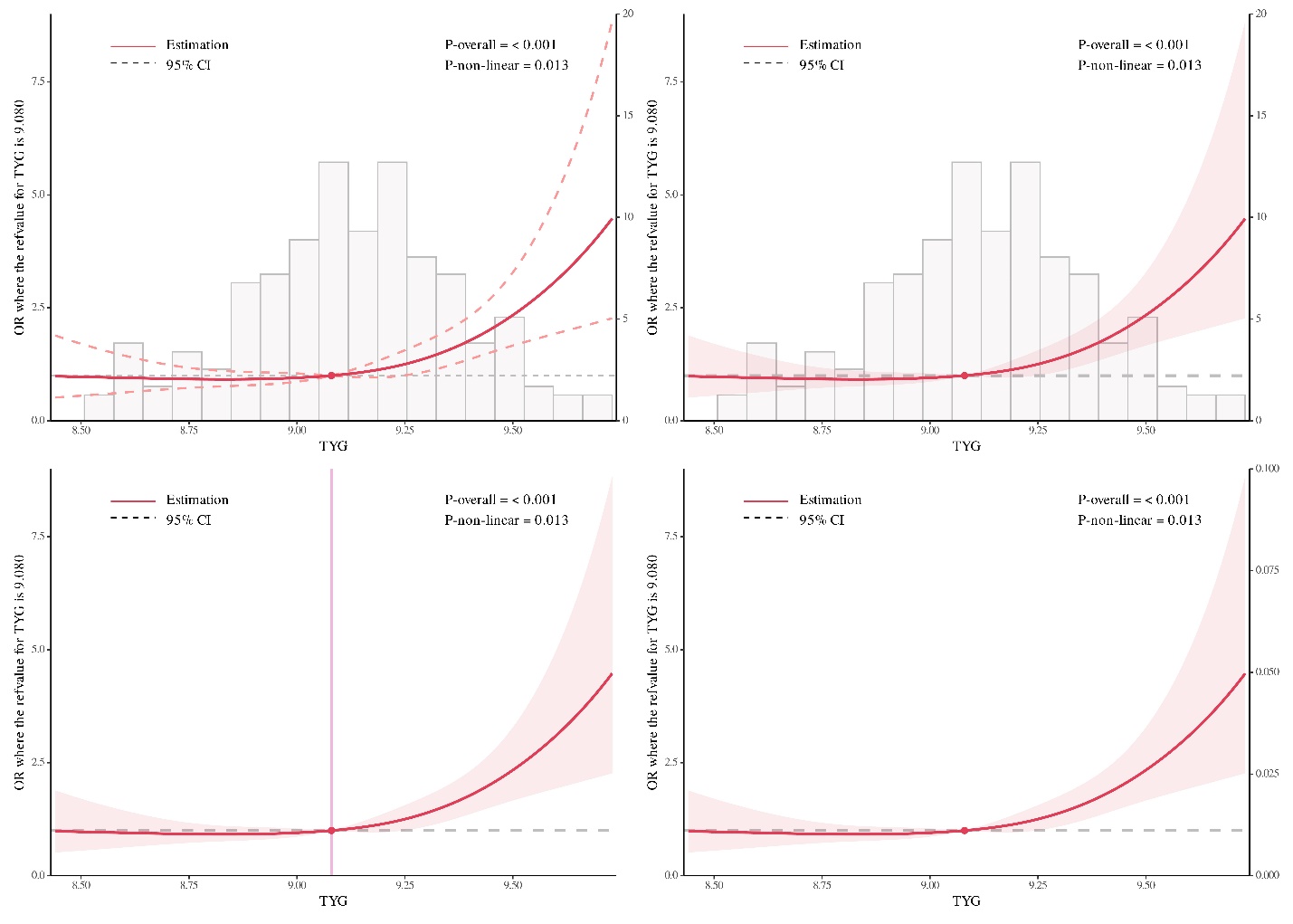


Figure S1 J-shaped associations between the TyG index and MAU after propensity score matching (PSM).


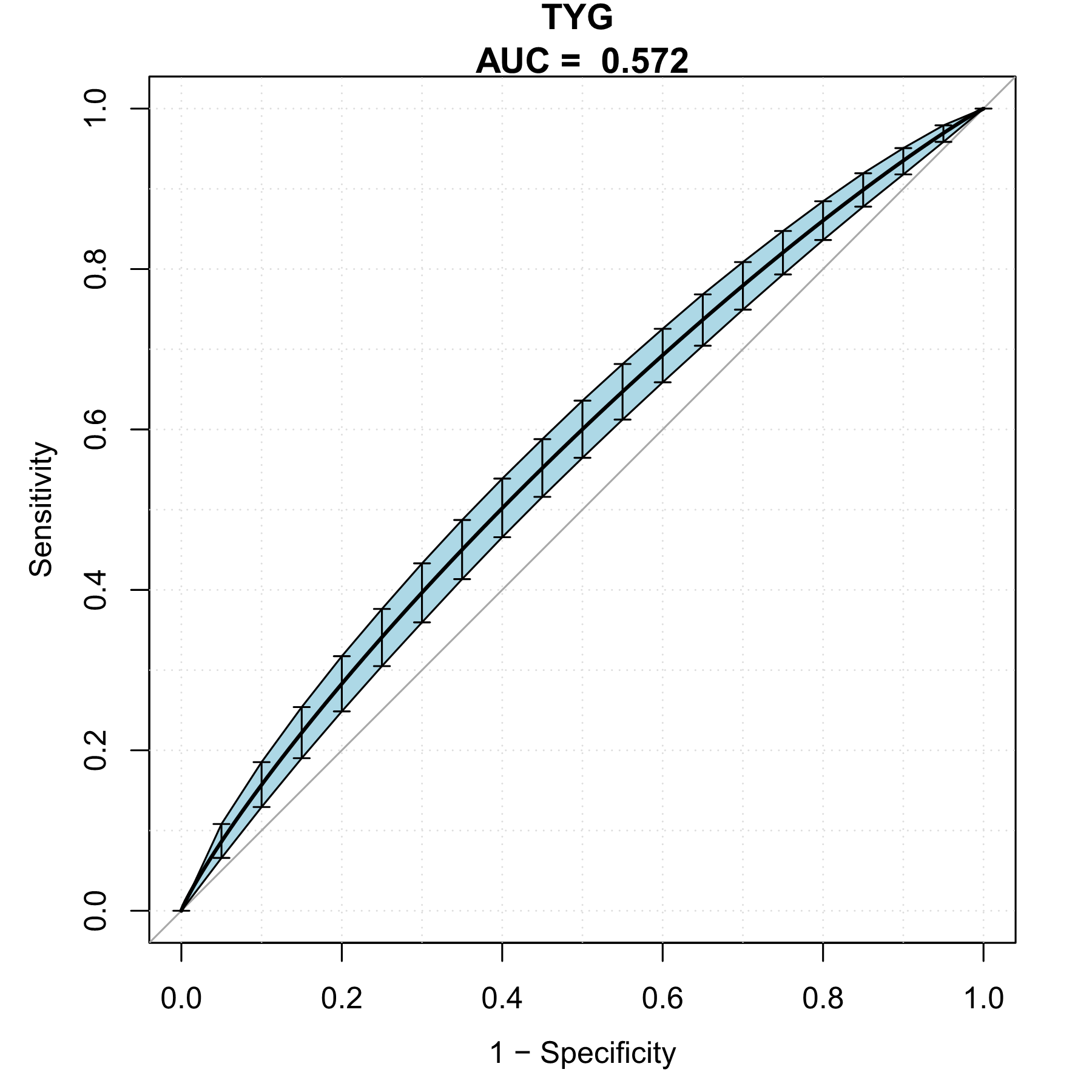


Figure S2 ROC curve of the TyG index for identifying microalbuminuria (MAU) in the original population. AUC = 0.572 (95% CI 0.545–0.593).


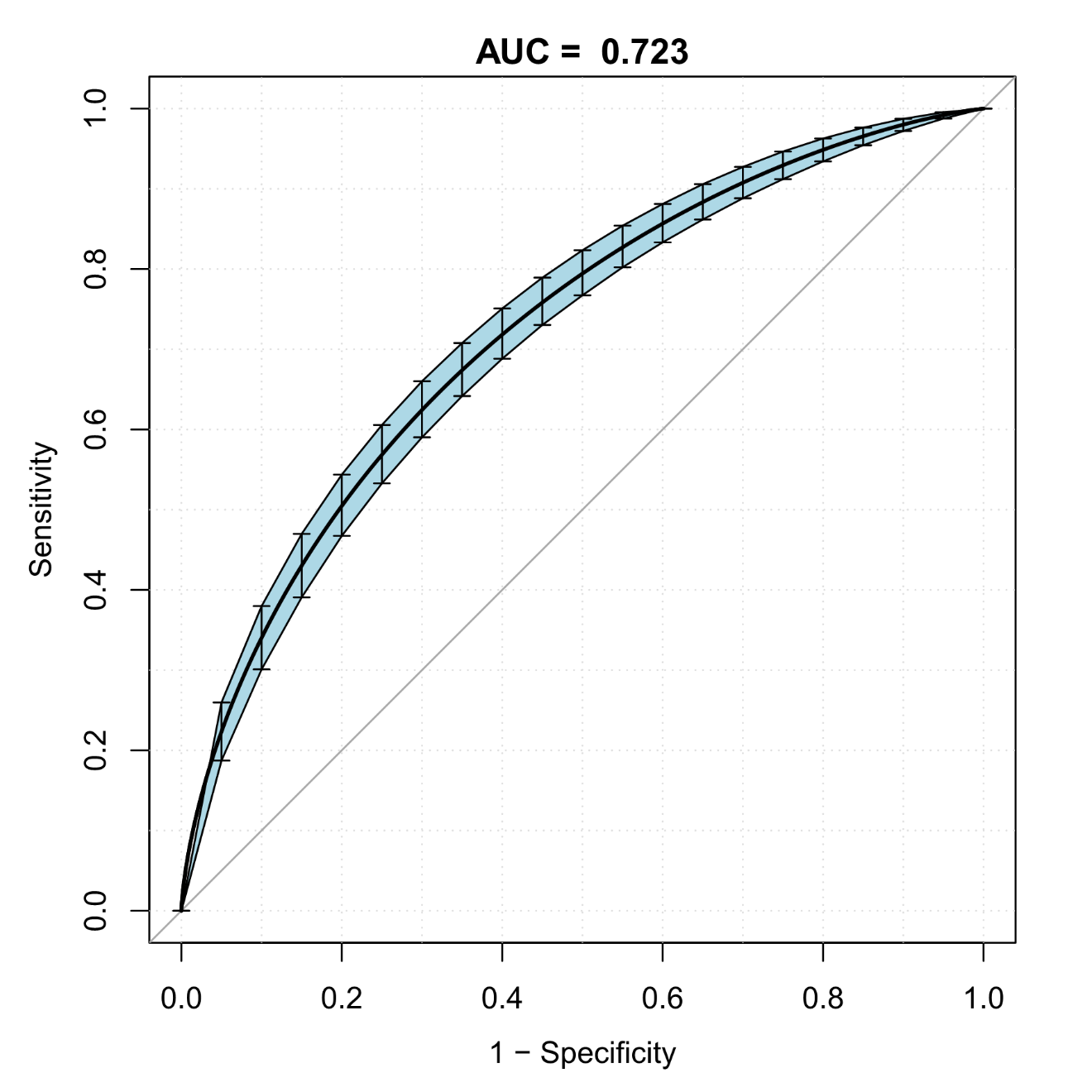


Figure S3 ROC curve of the TyG index for identifying MAU in the propensity score–matched (PSM) population. AUC = 0.723 (95% CI 0.701-0.745).
